# Supplementary material for: Patterns of Intron Gain and Loss in Fungi
Source: PLoS Biol. 2004 Nov 30;2(12):e422. doi: 10.1371/journal.pbio.0020422 (PMC532390; doi:10.1371/journal.pbio.0020422)
Supplement: Table S1 — Also available at http://genes.mit.edu/NielsenEtAl/. (4.3 MB ZIP). [file pbio.0020422.st001.zip › NielsenEtAl/html/857.html]

AN7252.1.NCU00455.1.MG05199.1.FG04101.1


```
 CLUSTAL W (1.82) Multiple Sequence Alignments - Introns Inserted


Sequence 1: MG05199.1	489 aa
Sequence 2: FG04101.1	492 aa
Sequence 3: NCU00455.1	480 aa
Sequence 4: AN7252.1	494 aa
Alignment Length: 509 aa
Number Identitical Residues: 198 aa
Alignment Score (without introns) 10170


MG05199.1 	-MSFNTGTAYAESDADDEYERDIHDSSPIDATDAEASPTESDP-PSNEHTPTTYGYRSSA
NCU00455.1	-MNFDSGTAYAESDADDEYERDIHGSSPIDHTDVEHSPIDSGP-PSAEHTPTTYGYRNSA
FG04101.1 	MMSFDGGTAYAESDADDEYERSMGDHSPI--TNSEHSPIDSELSTSAEHTPTTYGHRSSA
AN7252.1  	-MSLHT-SYHADSDADDEYERSVIT-SPHLATDSEGSASESEF-PSAEPTP-TFAHNEDN
          	 *.:.  : :*:*********.:   **   *: * *. :*   .* * ** *:.:... 

MG05199.1 	DRLPETIISEWTADECADFIATIGLEQYSTRFVE1NEIVGEALVALLHDDLKSMGIHSVG
NCU00455.1	DRHIETLISDWSADDCADFIASIGLQQYADRFIE1NEIVGEALVALQHDDLKSMGINSVG
FG04101.1 	DRLPETIITEWTVEECADFIGTIGLPQYADRFME1NEIVGEALVALQHDDLKSMGIASVG
AN7252.1  	SKIPDTIITEWTAEECASFVASLGLRQYCQAFIE1NGIVGEALIALRHDELKEMGIASVG
          	.:  :*:*::*:.::**.*:.::** **.  *:* * ******:** **:**.*** ***

MG05199.1 	HRLTILRSVYDVKKAQDVPVESDHYVPLT1AENEGQYATA--KDIKNLVEQLRLRDERMH
NCU00455.1	HRLMILKSVYDVKKAQDVPIEDDHYMPLT1AATEALSANATLNDIKHLAEQLRLRDERMS
FG04101.1 	HRLTILKSVYDVKKAQDVPMESDHYLPLS1ADAEAQYATATIKDIKHLVEQFRLRDERMS
AN7252.1  	HRLTILKSVYEMKVKQDIPLDADHYIPLS1AD-QSMNESATQEDIARLIQSIRLRDEKIM
          	*** **:***::*  **:*:: ***:**: *  :.   .*: :** .* :.:*****:: 

MG05199.1 	LFEQDFRRLAEDFRRLREDMLPALRLAKDAQQPLPHLNNGSAYAG-YDTTISPPAPTPST
NCU00455.1	LFEQDLRRLTEDFRRLREDMLPALRLAKDAQQPLP--SGPS-----YESTLSPPAPASSA
FG04101.1 	LLEQDLRRMTDDFRRLREDMLPALRLVKDAQQPLPNLSGNQQAYG-YETTISPPAPTPPP
AN7252.1  	TVETELRRVLEDYRRLREELLPVFKMAKDKSQPLPPPTAVSSSDGYYDDRLSGSTLTGQQ
          	 .* ::**: :*:*****::**.:::.** .****  .  .   . *:  :* .: :   

MG05199.1 	-----SGQSGGGLKRQWSTKKIMLGTTPKATSPTHLQTAHDRSLAEQTLDPSSAAERAVM
NCU00455.1	G----SQPSAG-LSRQYSTKRIMLGTTPKAVSPSHLQTSHG------PPYDSSASSRGML
FG04101.1 	G----SGQSGGSVKRQYSTRKILIGAAPKNASPT--QSTHERSIAETALDPTSAAERAVL
AN7252.1  	SGITILDRSGSILSRNLS-KRVHGGTTPKNNSPTHIPPSIHENRAYNDGTNLDPSAAAVA
          	.. :    *.. :.*: * :::  *::**  **:   .:   . :       ..:  .: 

MG05199.1 	SSSHLA-INGLVPSATSPSYPSINIPSPTSPPNT-LGGATLASRSYRSD-QPTPSSRSTF
NCU00455.1	SSSHLGALNGSSANTTSGGYPSPNMPSPTSPPTTNMSGTTLDPRSYRGDNQPPSSSRSTF
FG04101.1 	SSSHLAAMNGGGQGASSPSYPSPNIPSPTSPQTH-MSATTLASRSYRSE-APTPSSRTTF
AN7252.1  	ASSHLSTMNGKSQ-------LSPNLPSPTSPSNHYGAPQTLASRAYTQP-GSARNPHDHY
          	:****.::**           * *:****** .   .  ** .*:*     .. ..:  :

MG05199.1 	AESDYAHPG--GRDKQPVAP-RRMQTPAPDTP-SGSNASVEIFKSFRVSMDDPCYKVLPA
NCU00455.1	GDNDHYGSQ----DKQPVSAPRRGQTPAPDTPGSASNASVEIFKSFRVSMDDPCYKVLPA
FG04101.1 	NDDGHANAAYTSREKAPQGPPRRRETPVPDTP-SQSNASVEIFKSFRVSMEDPCYKVLPA
AN7252.1  	DDNPQSQSRSDRLNTTSSSATRSEIPSRSESRAGGETPSVEIFKSFRVSVDDPCYKVLPA
          	 :.    .     :. . ...*   .. .:: .. ...***********::*********

MG05199.1 	ALKKYQINAPWDQYALYIVYGDQERCLGLEEKPLILFKQLDKEGKKPMFMLRKTNNAQVD
NCU00455.1	ALKKYQINAPWDQYALYIVYGDQERCLGLDEKPLILFKQLDKEGKKPMFMLRKTNNAPAE
FG04101.1 	ALKKYQINAPWDQYALYIVYGDQERCLGLEEKPLILFKQLDKEGKKPMFMLRKTTTAPLD
AN7252.1  	ALKKYNINGDWKQYALYIVYGDQERCLGLEERPLILFKQLEKEGRKPMFMLRKQQQAPES
          	*****:**. *.*****************:*:********:***:********   *  .

MG05199.1 	IGNE--APGSAGLGS-ARGAATGYDPPGGII
NCU00455.1	E-----QPGSAGLSAAARGATTVYDPPGGII
FG04101.1 	G-----EPGSAGLGSTSRGAPTGYDPPGGII
AN7252.1  	TTAYPPSGGSAPNSAGFEPRQAQINLPGGVL
          	    ..  ***  .:  .   :  : ***::
```
